# Analysis of Multi-Tendon Friction-Based Composite Anchorage Device for CFRP Cables and Its Anchorage Mechanism

**DOI:** 10.3390/ma15082895

**Published:** 2022-04-15

**Authors:** Wanxu Zhu, Hongbin Chen, Wei Wei, Boxuan Chen, Xuejun Chen

**Affiliations:** 1 College of Civil and Architecture Engineering, Guilin University of Technology, Guilin 541004, China; zhuwanxu@vip.163.com (W.Z.); tcchb0325@163.com (H.C.); vvkitty1997@163.com (W.W.); chenbx1996@163.com (B.C.); 2 Collaborative Innovation Center for Exploration of Hidden Nonferrous Metal Deposits and Development of New Materials in Guangxi, Guilin 541004, China; 3 Guangxi Engineering Research Center of Intelligent Structural Material, Guilin University of Technology, Guilin 541004, China; 4 Guangxi Key Laboratory of Geotechnical Mechanics and Engineering, Guilin University of Technology, Guilin 541004, China

**Keywords:** CFRP cable, anchorage device, oblique cone, anchorage efficiency coefficient

## Abstract

Carbon fiber-reinforced polymer (CFRP) cables are anticipated to be employed in larger, longer, and more durable structures in the engineering field. However, its anchorage devices and mechanism should be appropriately developed and improved. At present, mainly relying on the adhesive force, most anchorage devices may lose their efficiency because of adhesive aging and failure or the slip of an individual tendon. A friction-based composite anchorage device with an integrated bearing of inner cone filler (i.e., load transfer media (LTM)) bonding and a single extruding anchor is proposed, and the anchorage mechanism is examined for Φ7 CFRP cables of strength 2400 MPa. Firstly, sufficient conditions for anti-slip failure of CFRP tendons in the anchorage zone are derived by assuming uniform LTM bonding. The obtained results reveal that the smaller inner pore size of the barrel leads to higher efficiency. Additionally, the maximum efficiency depends on the friction coefficient of the contact surface, the inner cone angle of the barrel, and the diameter and quantity of the CFRP tendons. The necessary conditions for the safety of the CFRP tendon anchorage zone are carefully obtained based on the Tsai–Wu failure criterion. It is concluded that the compressive stress of CFRP tendons in the anchorage zone should gradually increase from the load-bearing end to the no-loading end. Additionally, the relations among the anchorage efficiency coefficient and the CFRP tendon diameter *d*, the anchorage length *l*, the dip angle of LTM external conical surface *α*, and the friction angle *β* are derived based on the equivalent failure principle. The CFRP cables of four specifications (i.e., with Φ12, Φ19, Φ37, and Φ121 tendons) are designed under theoretical guidance, and eight static tests are carried out for more verification studies. The test results indicate that the anchorage efficiency coefficient of designed anchorage devices can be over 90%, and even up to 96.8%. Further, the failure modes are divergent destruction, which verifies the reliability of friction-based anchorage devices and provides a solid theoretical foundation for the design and engineering applications of CFRP cables.

## 1. Introduction

Permanent ground anchor reinforcement works are frequently exploited in extremely severe environments [1]. The safety and service life of engineering structures are seriously affected by the corrosion degradation, vibration fatigue, or other problems of conventional steel anchor cables [2], resulting in high maintenance costs [3]. Carbon fiber-reinforced polymer (CFRP) cables have been successfully employed in ground anchor reinforcement works [4] because of their excellent properties, such as lightweight, high strength, and corrosion/fatigue resistance [5], representing a broad development prospect in the engineering field. Nevertheless, there is no mature anchorage device system due to poor shear capacity and difficulties in the anchorage of CFRP tendons, significantly restricting the promotion and application of CFRP cables.

As a key element of the whole ground anchor system [6], anchorage devices can be classified into four types by anchorage mechanism, i.e., wedge-type, extruding, bond-type, and composite anchorage devices [7]. The wedge-type anchorage devices have been essentially improved by changing the cone angle of the inner anchor ring, the cone angle difference between the inner anchor ring and wedge, the preload of the wedge, and the length of anchorage devices [8,9]. Zhu W X. et al. [10] researched and developed a specific wedge-type anchorage device with an anchorage efficiency coefficient of over 90% and systematically studied the bridge reinforcement and engineering application. Zhu G P. [11] designed a wedge-type anchorage device with an anchorage efficiency coefficient of up to 94.9%. However, the wedge-type anchorage devices would be most applicable to single-tendon anchorage rather than multi-tendon anchorage, which inevitably damages CFRP tendons. The extruding anchorage devices are also not applicable to multi-tendon anchorage since the stiffness mutation between the anchorage device and tendons may easily cause damage to CFRP tendons with weak transverse strength [12]. At present, bond-type anchorage devices have been widely exploited for FRP multi-tendon anchorage. Scholars in China and abroad [13,14] have conducted a significant number of investigations on the adhesive (filler), radial compressive stress on the anchorage surface, fatigue performance, and stress relaxation of bond-type anchorage devices. Heeyoung et al. [15] found that the adhesive force of the anchorage device increases by lessening the sleeve’s diameter. Mei K H et al. [16] developed a particular bond-type anchorage device with a straight tube and inner cone, in which the stress concentration on the load-bearing end can be decreased by reducing the size of the anchorage device. Bo Feng et al. [17] proposed a bond-type anchorage device for CFRP cables with an anchorage efficiency coefficient of up to 97%. To this end, the load transfer media (LTM) with different stiffness are implemented to lessen the stress concentration. Wang et al. [18] researched and developed a winding cone anchorage system with variable stiffness whose anchorage efficiency coefficient was reported to be over 100%. Many studies have revealed that the bond-type anchorage devices are also applicable to multi-tendon anchorage by causing little damage to tendons [19]. However, if exposed to stresses, condensate water [20], temperature differences, acid, alkali, and other complex service environments in the long term, the adhesive force between the tendons and adhesive [21] will be gradually decreased or even lost. Additionally, for the bond type anchorage device of large-tonnage multi-tendon cables, the anchorage efficiency coefficient of CFRP cables will drastically reduce with the increase of tendons. The whole cables are suddenly broken due to the slip of inner ring tendons [22], making it difficult to effectively anchor the CFRP cables with a large diameter and tension [23]. Multi-tendon CFRP cables cannot be reliably anchored by a single anchorage type, promoting studies on composite anchorage devices. The performed investigations showed that for the wedge- and bond-type composite anchorage devices, the slip of CFRP tendons can be reduced by increasing the radial compressive stress of the contact surface, thus improving their ultimate bearing capacity [24,25]. However, such devices are still anchored by adhesive forces, with uncertain and unproven durability. It should be noticed that these devices are not extensively employed in engineering applications due to their commonly large dimensions.

In the present work, the application of the multi-tendon composite anchorage device for CFRP cables integrating inner cone filler bonding and single tendon extruding is proposed. It is envisaged that CFRP tendons can be reliably anchored in the presence of the synergistic effect of friction force generated by compressing and bonding the inner cone filler and the bearing of a single extruding anchor. Major emphasis is placed on the scrutiny of factors influencing the stress distribution of tendons and corresponding influence rules to reveal the anchorage mechanism of this particular anchorage device. Furthermore, this paper aimed to propose solutions for solving the anchorage problem of multi-tendon CFRP cables and to provide theoretical basis and technical support for the anchorage device design.

## 2. Theoretical Analysis of Multi-Tendon Friction-Based Anchorage Devices for CFRP Cables

Slip, pinch-off, and divergent destruction are three typical damage types for CFRP tendon anchorage devices. Slip or pinch-off of the anchorage device indicates that the overlarge relative displacement or compressive stress of the CFRP tendon in the anchorage zone has resulted in anchorage failure. The divergent destruction shows that the internal stress distribution of the anchorage device is reasonable, and the tendon tensile strength is fully exerted, which demonstrates the ideal anchoring effect. By this view, the emphasis of this study is to examine the influence of crucial parameters of the anchorage device on the stress distribution of the CFRP tendon in the anchorage zone. The chief goal is to guarantee that the tendon is clamped without excessive damage and achieve an efficient anchorage system.

### 2.1. Overall Scheme of the CFRP Friction-Based Anchorage Device

**Figure 1:**
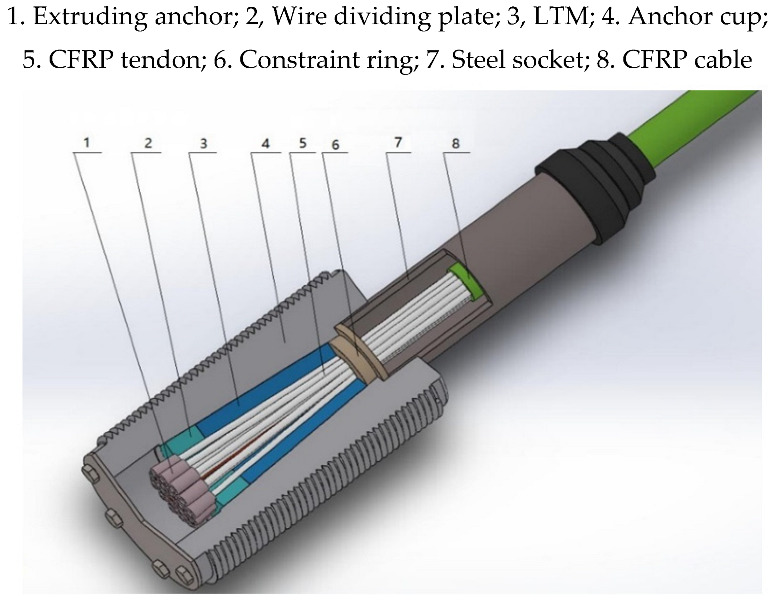
Composite anchorage device.

According to the research results, a high-performance steel cable [26] device for CFRP cables, integrating inner cone filler bonding and single tendon extrusion, is proposed (Figure 1). The extruding anchor is mechanically pressed at the end of each CFRP tendon and supported by a wire dividing plate. After LTM perfusion and curing, the extruding anchor and the inner cone packing are tightly connected in series by excessive compression. When the extruding anchor pushes the wire dividing plate to press the LTM tightly on the CFRP tendon, the CFRP tendon is anchored by the synergistic effect. The LTM can be made of cement, resin, resin-metal mixture, or fiber [8]. If the critical sliding friction resistance between the LTM and the CFRP tendon is greater than the cable force through a rational design of the anchorage device dimensional parameters, the adhesive force between them and the load sharing of the extruding anchor can be neglected.

### 2.2. Anti-Slip Conditions for the CFRP Anchorage Device

**Figure 2:**
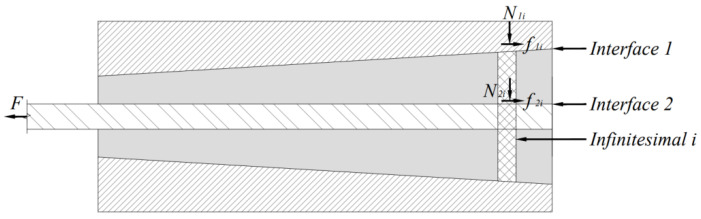
Stress analysis for composite anchorage device.

As demonstrated in Figure 1, under compression, friction appears between CFRP tendons in the anchorage device and the LTM as well as between the LTM and the anchorage device. It is assumed that in any section of the oblique cone, each CFRP tendon is dispersed in the LTM. Due to the different force conditions of consisting parts of the oblique cone, formed by the LTM and tendons, a rational strategy should be followed in the analysis. To this end, the anchorage device is appropriately divided into *n* sections from the load-bearing end along the direction of the tendon, and as a sample, the micro-unit of the *i* th section (*i* = 1,2,…,*n*) is taken into account for stress analysis (see Figure 2).

**Figure 3:**
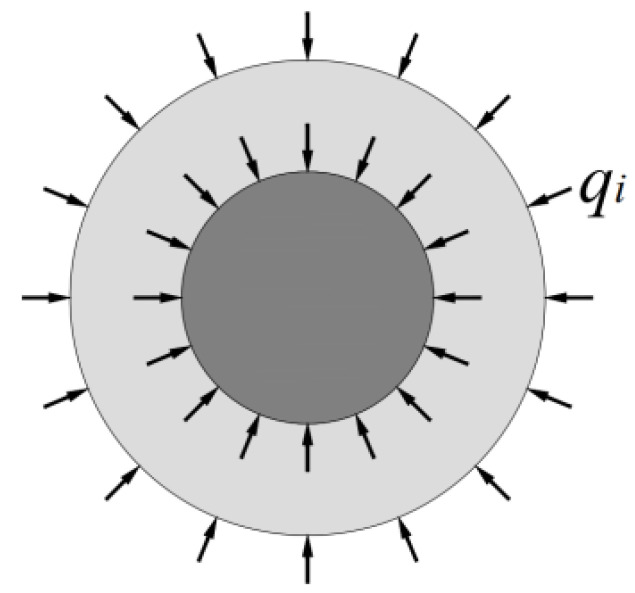
Stress analysis for cross section.

According to Figure 3, the LTM and CFRP tendons integrate when the CFRP composite anchorage device is over-compressed. Under such a circumference, these main constituents are subjected to the same radial compression as the LTM, precisely equal to that at the interface of the LTM and the barrel inner cavity. By the force analysis of the tendons and the LTM as a whole integrated system, the following results can be drawn:

The interface friction resistance between the barrel inner cone and the LTM of any micro-unit *i*:

(1) $$\begin{array}{l} f_{1i}=N_{1i}\cdot \sin\alpha +\mu \cdot N_{1i}\cdot \cos\alpha \\ f_{1i}=\left(q_{i}\cdot \cos\alpha \cdot \frac{D_{i}\pi \Delta l}{\cos\alpha }\right)\cdot \sin\alpha +\mu \cdot \left(q_{i}\cdot \cos\alpha \cdot \frac{D_{i}\pi \Delta l}{\cos\alpha }\right)\cdot \cos\alpha \\ f_{1i}=\left(\mu_{1}\cos\alpha +\sin\alpha \right)D_{i}q_{i}\pi \Delta l \end{array}$$

where *f*_1*i*_—the frictional force applied to interface 1.

*α*—the dip angle of the external conical surface of the oblique cone.

*μ*_1_—the friction coefficient of the interface between the LTM and the barrel inner cone hole for any micro-unit.

*N*_1*i*_—the positive pressure of the interface between the LTM and the barrel inner cone hole for the *i*th micro-unit.

*D_i_*—barrel inner cone size of the section of the *i*th micro-unit.

*q_i_*—radial compression applied to the *i*th micro-unit.

Δl—length of the consisting micro-units.

Friction resistance between the tendons and the LTM for the *i*th micro-unit.

(2) $$f_{2i}=\mu_{2}N_{2i}=\mu_{2}cdq_{i}\pi \Delta l$$

where *f*_2*i*_—the frictional force applied to interface 2.

*μ*_2_—the friction coefficient of the contact surface between the tendons and the LTM for any micro-unit.

*N*_2*i*_—positive pressure on the contact surface between the tendons and the LTM of the i th micro-unit.

*d*—diameter of the single CFRP tendon. *c*—number of CFRP tendons.

To ensure that the slip damage of CFRP tendons is not generated from the LTM, the friction resistance between the tendons and the LTM of each micro-unit must be more than the friction between the LTM and the barrel inner cone hole, i.e., $f_{2i}\geq f_{1i}$, which yields:

$$\mu_{2}cdq_{i}\pi \Delta L\geq \left(\mu_{1}\cos\alpha +\sin\alpha \right)D_{i}q_{i}\pi \Delta L$$

$$D_{i}\leq \frac{\mu_{2}}{\mu_{1}\cos\alpha +\sin\alpha }cd$$

As the LTM has a specific transverse stiffness, the closer the tendons to the center, the less the radial compression suffered. Therefore, a radial stress inhomogeneity coefficient *ψ* (0 < *ψ ≤* 1) is introduced, and its value can be determined by performing tests or finite-element analysis. Subsequently, the sufficient condition for slip damage in the CFRP tendon composite anchorage device may not be satisfied.

(3) $$D\leq \frac{\mu_{2}}{\mu_{1}\cos\alpha +\sin\alpha }cd\psi$$

where *D*—is the maximum inner pore size of the barrel.

Based on Equation (3), the inner pore size of the barrel should be as small as possible to guarantee the anti-slip failure of the CFRP tendon. Further, the maximum value of such a size relies on various factors, including the friction coefficient of the two contact interfaces, the inner cone angle of the barrel, the tendon diameter, and the quantity of CFRP tendons.

### 2.3. Necessary Conditions for the Non-Failure of the Anchorage Device

The common strength theories for composite materials are the maximum stress, the maximum strain, the Tsai–Hill strength, and the Tsai–Wu tensor criteria. The latter is based on the tensor function theory, which is particularly effective in modeling the mechanical behavior of anisotropic materials [27]. This fact is mainly attributed to its leading characteristic to include invariant theories consisting of tensors and tensor function structures with specific properties [28]. By this virtue, the Tsai–Wu failure criterion would be more suitable for investigating the mechanical response and strength of the orthotropic carbon fiber tendons. The general formula of this criterion is as follows:

(4) $$F_{i}\sigma_{i}+F_{ij}\sigma_{i}\sigma_{j}\ldots =1(i,j=1,2,\ldots ,6)$$

Assuming that each CFRP tendon experiences an axisymmetric stress state within the anchorage device. Equation (4) could be simplified as:

(5) $$F_{1}\sigma_{1}+F_{2}\sigma_{2}+F_{6}\sigma_{6}+F_{11}\sigma_{1}^{2}+F_{22}\sigma_{2}^{2}+F_{66}\sigma_{6}^{2}+2F_{16}\sigma_{1}\sigma_{6}+2F_{26}\sigma_{2}\sigma_{6}+2F_{12}\sigma_{1}\sigma_{2}$$

In which $\sigma_{1}$, $\sigma_{2}$, and $\sigma_{6}$ represent the axial stress, radial stress, and shear stress, respectively.

Where $F_{i}$, $F_{ij}$ are the strength tensors of the material. These two describe the interactions between the *i* th and *j* th principal stresses, obtained by tests and calculated based on the following formulas:

(6) $$\left\{ \begin{array}{l} F_{1}=\frac{1}{X_{t}}-\frac{1}{X_{c}},F_{11}=\frac{1}{X_{t}\cdot X_{c}}\\ F_{2}=\frac{1}{Y_{t}}-\frac{1}{Y_{c}},F_{22}=\frac{1}{Y_{t}\cdot Y_{c}},F_{12}=\frac{1}{2\sigma_{m}^{2}}\left[1-\left(\frac{1}{X_{t}}-\frac{1}{X_{c}}+\frac{1}{Y_{t}}-\frac{1}{Y_{c}}\right)\sigma_{m}-\left(\frac{1}{X_{t}X_{c}}+\frac{1}{Y_{t}Y_{c}}\right)\sigma_{m}^{2}\right]\\ F_{16}=F_{26}=F_{6}=0,F_{66}=\frac{1}{S^{2}} \end{array}\right.$$

where $X_{t}$ and $X_{c}$ denote the axial tensile and compressive strength, $Y_{t}$ and $Y_{c}$ are the radial tensile and compressive strength, respectively, and S is the shear strength. For high-strength CFRP tendons, $X_{t}$ is determined for the carbon fiber yarns. Based on the strength of engineering applications, it is obtained $X_{t}$ = 2400 MPa. Other factors, $X_{c}$, $Y_{t}$, and $Y_{c}$, are determined for isotropic epoxy resin, and based on the performed tests, $X_{c}$ = $Y_{t}$ = $Y_{c}$ = 120 MPa are obtainable. By substituting these factors into Equation (6), it is readily evaluated that $F_{2}$ = 0. The radial tensile strength was remarkably lower than the axial tensile strength; hence, one can rationally write: $\sigma_{m}\approx -Y_{t}=-X_{c}$. Additionally, the radial and axial directions of the CFRP tendons are basically the same as those of the principal stresses in the anchorage device, and thereby, it is observable: $\sigma_{6}$ = 0. The simplified expression for $F_{12}$ in Equation (6) is given by:

(7) $$F_{12}=\frac{1}{2X_{c}^{2}}-\frac{1}{X_{t}X_{c}}$$

Equations (6) and (7) are substituted into Equation (5), and the resulting expression can be simplified as:

$$\left(\frac{1}{X_{t}}-\frac{1}{X_{c}}\right)\sigma_{1}+\frac{\alpha_{1}^{2}}{X_{t}X_{c}}+\frac{\alpha_{2}^{2}}{X_{c}X_{c}}+\frac{\alpha_{1}\alpha_{2}}{X_{c}^{2}}\left(1-2\frac{X_{c}}{X_{t}}\right)=1$$

(8) $$\frac{1}{X_{c}^{2}}\left(\frac{X_{c}^{2}}{X_{t}}-X_{c}\right)\sigma_{1}+\frac{X_{c}}{X_{t}}\alpha_{1}^{2}+\alpha_{2}^{2}+\alpha_{1}\alpha_{2}\left(1-2\frac{X_{c}}{X_{t}}\right)=1$$

Let $\zeta =\frac{X_{t}}{X_{c}}$, ζ=20. Furthermore, the compressive stress concentration coefficient is defined as: $\eta =\frac{\sigma_{2}}{X_{c}}$. By substituting these relations into Equation (8), one can write:

$$\frac{1}{X_{c}^{2}}\left(\frac{1}{\xi }-1\right)\sigma_{1}X_{c}+\frac{1}{\xi }\sigma_{1}^{2}+\eta^{2}\sigma_{2}^{2}+\eta \sigma_{1}X_{c}\left(1-\frac{2}{\xi }\right)=1$$

$$\frac{1}{\xi X_{c}^{2}}\left[\sigma_{1}^{2}+\left(1+\xi \eta -2\eta -\xi \right)\right]X_{c}\sigma_{1}+\xi \eta^{2}X_{c}^{2}=1$$

$$\sigma_{1}=X_{c}\left[\sqrt{\frac{{\left(1-\xi -\eta \xi \right)}^{2}}{4}+\xi \left(1-\eta^{2}\right)}-\frac{1-\xi -\eta \xi }{2}\right]$$

(9) $$\sigma_{1}=0.05X_{t}\left[\sqrt{\frac{{\left(19+20\eta \right)}^{2}}{4}+20\left(1-\eta^{2}\right)}-\frac{19+20\eta }{2}\right],\eta \in \left[-1,0\right],\sigma_{1}\in \left[0,2400\right]$$

MATLAB software is implemented to simplify Equation (9) as obtained in the following:

$$\sigma_{1}-19.06\sigma_{2}=X_{t}$$

Then the necessary conditions for the safe state of CFRP tendons in the anchorage device would be:

(10) $$\sigma_{1}-19.06\sigma_{2}\leq X_{t}$$

In other words, the stress distribution of each CFRP tendon in the anchorage device is restricted by the given condition in Equation (10), and the tendon failure can be avoided if the axial and radial stresses meet this relation.

### 2.4. Stress Adjustment Mechanism in the Anchorage Device

The anchorage efficiency coefficient, *η_A_*, is commonly employed to measure and evaluate the performance of the anchorage device. The greater the value of the aforementioned coefficient, the better the anchorage property (i.e., the ratio of the actual tensile force to the standard ultimate tensile force of the CFRP tendons at the time of damage). In other words,

(11) $$\eta_{A}=\frac{F_{pk}}{F_{b}}$$

where *F_pk_* represents the ultimate breaking force of the anchorage device, and *F_b_* is the standard ultimate tensile force of the CFRP tendons.

**Figure 4:**
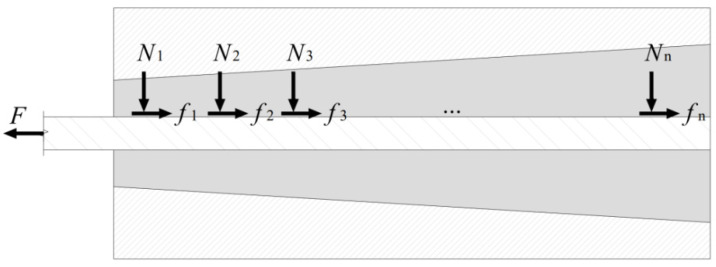
Stress analysis for oblique cone.

According to Figure 4, the anchorage device is subdivided into *n* segments from the loaded section in the direction of the tendon, subjected to the radial stress $N_{i}$ as well as the axial friction force $f_{i}$.

Due to unfavorable factors, such as the part inhomogeneity of the oblique cone, a stress concentration from the axial tension and radial pressure is induced in the *i*th micro-unit:

(12) $$\sigma_{1}=k_{i1}\bar{\sigma_{1i}},\sigma_{2}=k_{i2}\bar{\sigma_{2i}},i=1,2,3,\ldots ,n$$

where $\bar{\sigma_{1i}}$ and $\bar{\sigma_{2i}}$—the average tensile and compression stresses within the *i*th micro-unit section of the CFRP tendon, respectively.

$k_{i1}$ and $k_{i2}$—the tensile and compression stress concentration coefficients of the *i*th micro-unit section of the CFRP tendon, respectively.

Using Equation (10), the relationship between the tensile stress and compression one in the *i*th micro-unit section within the anchorage device in a safe state should be in accordance with the following formula:

(13) $$k_{i1}\bar{\sigma_{1i}}-19.06k_{i2}\bar{\sigma_{2i}}\leq X_{t}$$

Assuming that the stress concentration coefficients for various micro-unit sections would be the same (i.e., $k_{1}$, $k_{2}$), which are substituted into Equation (13), in the section micro-unit i:

(14) $$k_{1}\bar{\sigma_{1i}}-19.06k_{2}\bar{\sigma_{2i}}\leq X_{t}$$

To make the tendons less prone to damage and fracture and achieve a high anchorage efficiency coefficient, the stress concentration zone of each section should be in an equivalent failure state, representing the ideal situation. By this view, we can state for any section micro-unit *i*, *j*:

(15) $$k_{1}\bar{\sigma_{1i}}-19.06k_{2}\bar{\sigma_{2i}}=k_{1}\bar{\sigma_{1j}}-19.06k_{2}\bar{\sigma_{2j}},i,j=1,2,3,\ldots ,n$$

From the small end of the barrel cone hole to the large end, the tensile stress of each section micro-unit gradually decreases by the axial friction resistance of the LTM to the CFRP tendon. The average tensile stress can be given by:

(16) $$\bar{\sigma_{1i}}=\frac{F}{S_{i}}-\frac{F_{1}}{S_{i}}-\frac{F_{2}}{S_{i}}-\ldots -\frac{F_{\left(i-1\right)}}{S_{i}}=\frac{F-\sum_{m=1}^{i-1}F_{m}}{S_{i}}$$

where $S_{i}$ (*i* = 1,2,…,*n*) denotes the actual sectional area of the CFRP tendon. It should be noticed that the variation of the tendon’s sectional area, compressed by the LTM, is insignificant at all and could be neglected. As a result, the cross-section area of each tendon can be rationally recorded as *S*.

By substituting Equation (16) into Equation (15), it is obtainable:

(17) $$k_{1}\frac{F-\sum_{n=1}^{i-1}F_{n}}{S}-19.06k_{2}\frac{N_{i}}{S_{h}}=k_{1}\frac{F-\sum_{m=1}^{j-1}F_{m}}{S}-19.06k_{2}\frac{N_{j}}{S_{h}}$$

where $S_{h}$ is the side area of the *i*th micro-unit section, thereby, $S_{h}=\frac{l}{n}\pi d$, in which *l* represents the effective anchorage length.

It is simplified to obtain:

(18) $$k_{1}\left(F-\sum_{n=1}^{i-1}F_{n}\right)-\frac{4.765k_{2}nd}{l}N_{i}=k_{1}\left(F-\sum_{n=1}^{j-1}F_{n}\right)-\frac{4.765k_{2}nd}{l}N_{j}$$

As a general trend, the closer the section to the load-bearing end, the larger the value of $F-\sum_{n=1}^{i-1}F_{n}$. Therefore,

(19) $$N_{n}>N_{\left(n-1\right)}>\ldots >N_{1}$$

As can be seen, the compression force from the load-bearing end to the no-loading end of the anchorage device, particularly the CFRP tendon, should be gradually increased to achieve a better anchorage property. To this end, specific technology or design of the detailed structure of the barrel inner pore can be implemented relation.

From the analysis given above and Equation (14), it can be obtained:

(20) $$\sum_{i=1}^{n}k_{1}\bar{\sigma_{1i}}S_{i}-19.06\sum_{i=1}^{n}k_{2}\bar{\sigma_{2i}}S_{i}\leq n\cdot X_{t}\cdot S$$

Let $k_{2}^{’}=\frac{4.765nd}{l}k_{2}$, it can be deduced that:

$$k_{1}\sum_{i=1}^{n}\left(F-\sum_{j=1}^{i-1}F_{j}\right)+k_{2}^{’}\sum_{i=1}^{n}N_{i}\leq nF_{b}$$

The above relation can be analyzed as follows:

(21) $$k_{1}\left(nF-\sum_{i=1}^{n}\sum_{j=1}^{i-1}F_{j}\right)+k_{2}^{’}\sum_{i=1}^{n}N_{i}\leq nF_{b}$$

Assuming that there exists a consistent direct proportional relationship between the extruding force *N_i_* and the friction resistance force *F_i_* for each section, i.e.,

(22) $$F_{i}=kN_{i}$$

where k is the proportionality coefficient, and β is the friction angle between the barrel and LTM.

(23) $$\sum_{i=1}^{n}F_{i}=k\sum_{i=1}^{n}N_{i}$$

Since $F=\sum_{i=1}^{n}F_{i}$, i.e.,

(24) $$F=k\sum_{i=1}^{n}N_{i}$$

Therefore:

$$\begin{array}{ll} \sum_{i=1}^{n}\sum_{j=1}^{i-1}F_{j} & =\left(n-1\right)F_{1}+(n-2)F_{2}+\cdots +2F_{n-2}+F_{n-1}\\ & =(\frac{n-1}{2})F_{1}+(\frac{n-2}{2}F_{2}+\frac{1}{2}F_{1})+(\frac{n-3}{2}F_{3}+\frac{1}{2}F_{2}+\frac{1}{2}F_{1})+\cdots +\sum_{i-1}^{n}\frac{1}{2}F_{i}\\ & =k\left[(\frac{n-1}{2}N_{1})+(\frac{n-2}{2}N_{2}+\frac{1}{2}N_{1})+(\frac{n-3}{2}N_{3}+\frac{1}{2}N_{2}+\frac{1}{2}N_{1})+\cdots +\sum_{i-1}^{n}\frac{1}{2}N_{i}\right] \end{array}$$

(25) $$\sum_{i-1}^{n}\sum_{j-1}^{i-1}F_{j}\leq \frac{n-1}{2}k\sum_{i=1}^{n}Ni=\frac{n-1}{2}F$$

By substituting Equations (24) and (25) into Equation (21), the following relation is derived:

(26) $$k_{1}F\left(n-\frac{n-1}{2}\right)+k_{2}^{’}\frac{F}{k}\leq nF_{b}$$

i.e.,

(27) $$\frac{F}{F_{b}}\leq \frac{n}{k_{1}\frac{n+1}{2}+\frac{k_{2}^{’}}{k}}$$

$k_{2}^{’}=\frac{4.765nd}{l}k_{2}$ was substituted to obtain:

(28) $$\frac{F}{F_{b}}\leq \frac{n}{k_{1}\frac{n+1}{2}+\frac{4.765ndk_{2}}{lk}}$$

as *n* approaches infinity, then:

(29) $$\frac{F}{F_{b}}\leq \frac{2lk}{lkk_{1}+9.53dk_{2}}$$

*F = F_pk_*, when *F* reaches the ultimate breaking force of the anchorage device, *F_pk_*, then:

(30) $$\eta_{A}=\frac{F_{pk}}{F_{b}}\leq \frac{2lk}{lkk_{1}+9.53dk_{2}}$$

k=ψtan(α+β) is substituted into the formula and summarized to obtain:

(31) $$\eta_{A}\leq \frac{2}{k_{1}+9.53\frac{dk_{2}}{l\psi \tan\left(\alpha +\beta \right)}}$$

According to Equation (31), anchorage efficiency coefficient *η_A_* depends on various factors, including the stress concentration coefficients *k*_1_ and *k*_2_, anchorage length *l*, single tendon diameter *d*, radial stress inhomogeneity coefficient *ψ*, the dip angle of the LTM external conical surface *α*, and its friction angle *β*. The smaller *k*_1_ and *k*_2_ were, the larger the *η_A_* was. The greater the sum of *α* and *β*, the greater the *η_A_*. The smaller the ratio of *d* to *l*, the greater the *η_A_*. The value of *η_A_* increases by reducing the value of *k*_1_, *k*_2_, *d*, or *l*; however, an increase of *α* and *β* leads to the growth of *η_A_*.

## 3. Determination of the Dimensional Parameters of the Anchorage Device

The crucial dimensional parameters of the anchorage device could be determined based on Equations (3), (18) and (31) in conjunction with the experiments through comparative studies. For this purpose, the following specific steps are suggested:

(1) Determination of the barrel parameters and LTM materials:

Since the barrel is subjected to high circumferential tensile stress with high safety requirements, it is made of alloy steel or stainless steel with high toughness and strength to meet the specific application process of the anti-corrosion requirements of the occasion. Generally, 40Cr steel can be employed with quenching and tempering treatments. For the LTM of our concern, epoxy iron sand filler with low transverse stiffness is selected, whose casting curing temperature should be appropriately controlled to avoid affecting the mechanical properties of the CFRP tendon.

(2) Determination of the inner pore cone angle of the barrel (*α*) and the diameter of the large end (*D*):

Generally, the value of *α* should be slightly smaller than the cold-cast barrel inner pore cone angle of 5° for steel cables to prevent the CFRP tendon from slip failure. The value of *α* = 3° would be appropriate for the test, which can be optimized based on the finite-element analysis and test results.

The friction coefficient of the contact surface under the action of high stress was determined by Huang Z N [29]. Herein, the friction coefficient between the LTM and the barrel is set equal to 0.23, i.e., *μ*_1_ = 0.23 for interface 1 and the friction angle *β* = 13°. When the friction coefficient between the LTM and the CFRP tendon would be 0.32, we have *μ*_2_ = 0.32, which is substituted into Equation (3) to obtain:

(32) D≤1.135cdψ

The value of *D* calculated by Equation (32) would be relatively small, and the LTM cannot be easily poured when making the multi-tendon anchorage device. Consequently, the synergistic bearing of the extruding anchor should also be taken into consideration. In other words, an increase in the friction coefficient of interface 2 leads to an increase in the maximum inner pore size of the barrel. In so doing, Equation (32) could be modified as follows:

(33) D≤1.135cdψ(1+γ)

where *γ* represents the proportion of the load sharing of the extruding anchor.

(3) Determination of the factor *l*

The length of the anchorage area should be as small as possible to lessen the size and weight of the anchorage device. When the value of *η*_A_ is considered greater than 0.9 according to the specification [30], the LTM is poured uniformly and densely in the anchorage zone. For such situations, the stress concentration can be taken into account in the calculations. It implies that the minimum values of $k_{1}$ and $k_{2}$ (1) are considered. Based on Equation (31), the minimum anchorage length of the CFRP tendon is evaluated as:

(34) $$l\geq \frac{9.53k_{2}d}{\left(\frac{2}{\eta_{A}}-k_{1}\right)\psi \tan\left(\alpha +\beta \right)}$$

(35) $$l\geq \frac{27.2d}{\psi }$$

The obtained value of *l* by Equation (35) would be relatively large, especially for cables with more tendons. Hence, the synergistic bearing of the extruding anchor should be considered by lowering the requirements of the anchorage efficiency coefficient. In doing so, Equation (35) could be modified in the following form:

(36) $$l\geq \frac{33.235}{\left(\frac{2}{\eta_{A}-\gamma }-1\right)\psi }d$$

(4) Trial design of the CFRP composite anchorage device

Four types of Φ7 CFRP tendon are selected for trial design in view of the strength of 2400 MPa for engineering practices and demands. The values of γ in order are taken as 0 and 0.2 for small tendons without and with considering the load sharing of the extruding anchor for the sake of safety. The larger the tendons are, the smaller the radial stress inhomogeneity coefficient *ψ* is, which is preliminarily set based on the experience. This factor can be optimized according to the finite-element analysis and test results. The maximum inner pore size of the barrel and the length limit can be obtained using Equations (33) and (36) (see Table 1).

**Figure 5:**
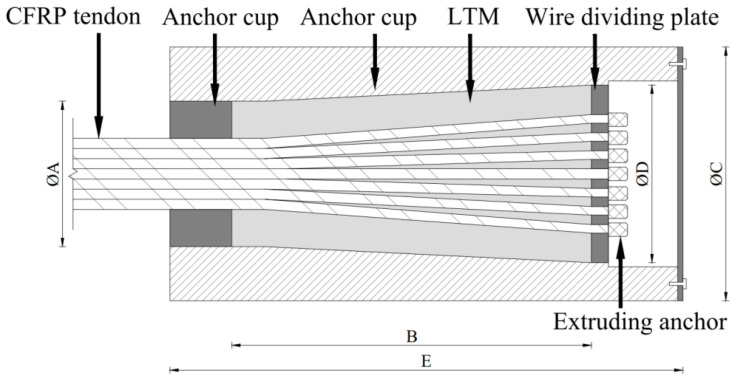
Φ7–37 Composite anchorage device.

According to Table 1, the inner pore of the barrel of 37 and 121 tendons is preliminarily set as a single cone. The schematic representation of the Φ7–37 CFRP anchorage device has been demonstrated in Figure 5. The specific design parameter of the anchorage device is also determined (see Table 2). Based on the finite-element method and test results, the radial compressive stress at the load-bearing end can be reduced through gradient cone angle, multi-cone, and LTM variable stiffness. This issue makes the CFRP tendon in the anchorage section closer to the equivalent failure state to achieve better anchorage properties.

## 4. Test Verification of the CFRP Composite Anchorage Device

**Figure 6:**
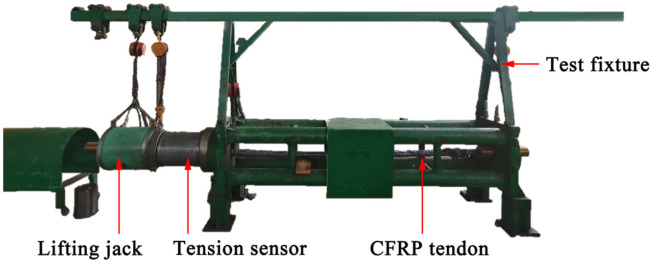
CFRP tendons and the test device.

The preliminary design of Φ7–12, Φ7–19, Φ7–37, and Φ7–121 CFRP cables are checked by static load tests in line with the requirements in the GB/T14370-2015 [30] and JGJ85-2010 specifications [31]. The tests are carried out in the structural experiment hall of the Guilin University of Technology (Guilin, China) and the testing center of Liuzhou OVM Machinery Co., Ltd. (Liuzhou, China) The length of the no-loading section of the test specimen is greater than 3 m. The test device has been presented in Figure 6.

**Figure 7:**
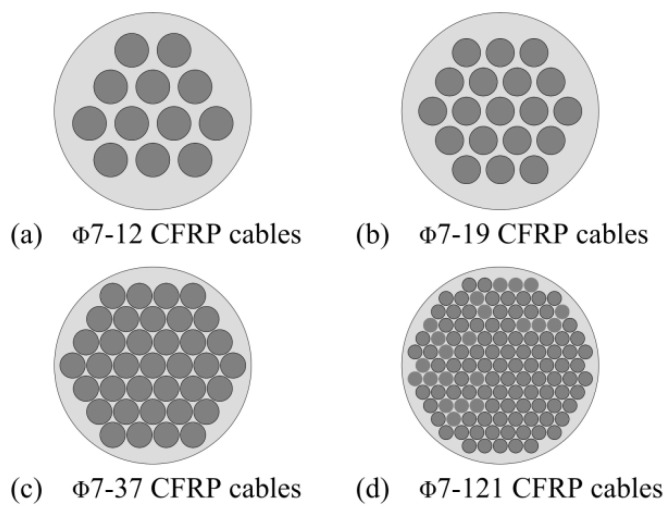
CFRP cables setup.

**Figure 8:**
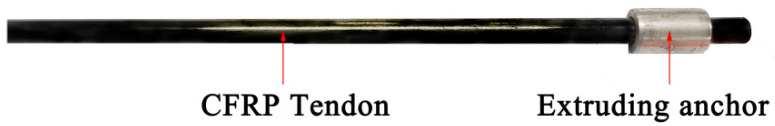
Assembly of CFRP tendon and extruding anchor.

The Φ7 plain CFRP tendon of strength 2400 MPa is chosen. The surface of the tendon has not been cleaned, and the residual release agent after pultrusion is retained to minimize the adhesive force between the tendon and the LTM. A total of eight sets of tests are carried out, including three sets of Φ7–12 and Φ7–19 CFRP cables and one set of Φ7–37 and Φ7–121 CFRP cables. The layout of the end of tendons has also been presented in Figure 7. The steel extruding anchor of diameter and length 12 and 20 mm, respectively, is extruded by extruding machine at the end of tendons (see Figure 8). The tensile breaking force of a single tendon and extruding anchor assembly is tested to be not lower than 20% of the tendon’s ultimate tensile strength.

**Figure 9:**
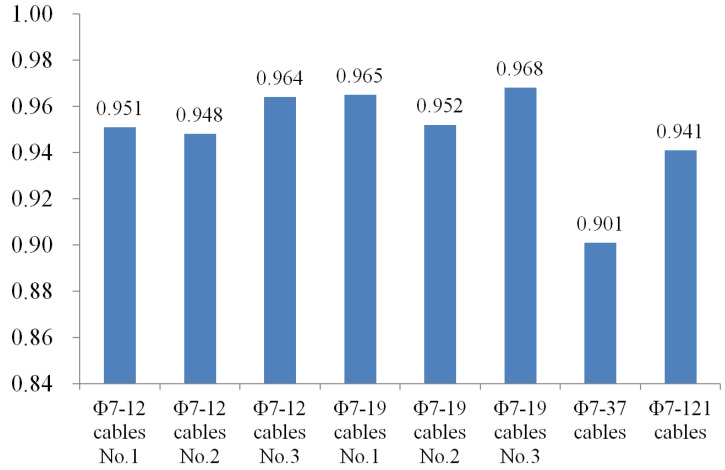
Static load tests result.

**Figure 10:**
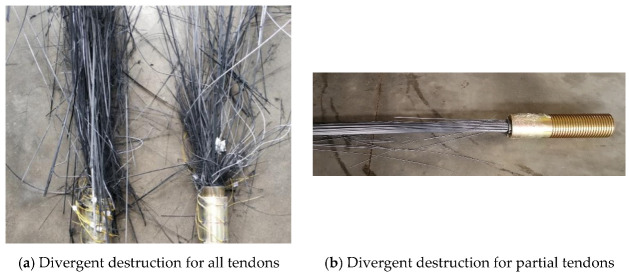
Typical damage types for CFRP cables.

The test was carried out with staged and graded loading. The graded loading at 10% *F_b_* is taken in the initial stage. The load is sustained for 30 min when it reaches 80%*F_b_* to test the load sustaining the stability of the anchorage device. Then it is changed to the graded loading at 5%*F_b_* until the specimen is damaged. The loading speed is also controlled at 200 Mpa/min during the loading. The results of the performed tests are shown in Figure 9. The tension damage appears in each group of tests, and the damage type of the CFRP tendon is divergent destruction (see Figure 10).

The test results (Figure 9) reveal that the anchorage efficiency coefficient *η*_A_ obtained in each group of tests is over 90%, taking its peak point at 96.8%. As presented in Figure 9, the failure state of the CFRP tendon represents divergent destruction, which is rather ideal. Notably, the determined size of the cone cup of the CFRP composite anchorage device can meet the anchorage requirements based on the theoretical analyses and calculations of the present work. The correctness of the proposed model and the reliability of the design are checked by the test results. The 37- and 121-tendon cables are at the limit state with only a few tendons in the failure state, and the anchorage efficiency coefficient is also lower. Therefore, it is required to optimize the structure and dimensional parameters through finite-element analysis.

## 5. Conclusions

The friction-based composite anchorage device integrating LTM bonding and single tendon extruding was proposed, and the corresponding anchorage mechanism was studied for Φ7 CFRP cables of strength 2400 MPa. The sufficient conditions for anti-slip failure of CFRP tendons in the anchorage zone were derived. By employing the Tsai–Wu failure criterion, the necessary conditions for the safety of CFRP tendons in the anchorage zone were also explained. The equivalent failure principle was proposed, and the relation between the anchorage efficiency coefficient and the influencing factors was obtained. The CFRP cables of four specifications (i.e., Φ12, Φ19, Φ37, and Φ121 tendons) were designed under the theoretical guidelines, and eight static load tests were carried out. The main obtained results are summarized as follows:

(1) The governing formulas of the anchorage efficiency coefficient *η*_A_, the stress concentration coefficients *k*_1_ and *k*_2_, the single tendon diameter *d*, the anchorage length *l*, the radial stress inhomogeneity coefficient *ψ*, the dip angle of wedge external conical surface *α*, and the friction angle *β* were obtained based on which CFRP tendon anchorage device was designed, and the corresponding minimum anchorage length was determined.
(2) The radial stress inhomogeneity coefficient *ψ* was proposed to carefully assess the sufficient conditions for the anti-slip failure of the CFRP tendon in the anchorage zone. The CFRP tendon could be prevented from slipping out of the anchorage device when the inner pore size of the barrel reaches the maximum value, and it should be as small as possible.
(3) The CFRP tendon should be gradually compressed tightly from the no-loading end to the load-bearing end; therefore, the stresses in the anchorage device could be reasonably distributed for better anchorage properties.
(4) Two types of CFRP tendon composite anchorage devices were preliminarily designed, and the static load tension tests were carried out under theoretical guidance. The anchorage efficiency coefficients of the designed anchorage devices could be over 90%, peaking at 96.8%, and the failure form of the two devices was divergent destruction, which was rather ideal. As a result, the theoretically designed anchorage device was reasonably verified with experiments.

## 6. Prospect

The results of this study can provide a solid basis and guidance for the design of the multi-tendon composite anchorage device for CFRP cables; however, the radial stress inhomogeneity coefficient *ψ* is preliminarily determined by experience. The finite-element calculations and anchorage strain test analyses of CFRP tendons with various specifications are then carried out to determine the more precise values. The performed examinations will provide practical guides for the design of such a type of anchorage device.

## Figures and Tables

**Table 1:** Parameters of the anchorage devices.

Quantity of the Φ7CFRP Tendons	γ	ψ	Maximum Diameter of the Large End *D* (mm)	Minimum Effective Anchorage Length *l* (mm)
12	0	0.8	76.27	237.93
19	0	0.75	82.63	253.79
37	0.2	0.7	246.93	178.96
121	0.2	0.3	346.08	415.44

**Table 2:** Parameters of anchorage devices.

Model of Anchorage Devices	Diameter of the Small End ΦA	Effective Anchorage Length B	Diameter of Anchorage Devices ΦC	Diameter of the Large End ΦD	Length of Anchorage Devices E
Φ7–12	42	254	72	53	280
Φ7–19	68	380	115	74	480
Φ7–37	86	193	150	105	303
Φ7–121	126	510	250	180	620

## Data Availability

All data, models, and code generated or used during the study appear in the submitted article.
